# Trends in body image of adolescent females in metropolitan and non-metropolitan regions: a longitudinal study

**DOI:** 10.1186/s12889-016-3815-1

**Published:** 2016-11-08

**Authors:** M. Craike, J. A. Young, C. M. Symons, M. D. Pain, J. T. Harvey, R. M. Eime, W. R. Payne

**Affiliations:** 1 Institute of Sport, Exercise and Active Living, College of Sport and Exercise Science, Victoria University, PO Box 14428, Melbourne, Victoria 8001 Australia; 2 School of Health Sciences, Federation University, PO Box 663, Ballarat, Victoria 3353 Australia

**Keywords:** Body image, Adolescent, Female, Longitudinal, Region, Body satisfaction, Adolescence

## Abstract

**Background:**

Body dissatisfaction is associated with a range of adverse outcomes, including impaired psychological health, low physical activity and disordered eating. This longitudinal study used the Factors Influencing Transitions in Girls’ Active Leisure and Sport (FITGALS) dataset to examine trends in body image of adolescent females. Specifically, the study examined satisfaction with body size, physical appearance and dieting behaviour for two cohorts at transitional life phases in two geographic regions longitudinally over a 3-year period.

**Methods:**

A sample of 732 adolescent females in Grade 7 (*n* = 489, 66.8 %) and Grade 11 (*n* = 243, 33.2 %) at randomly selected Australian metropolitan and non-metropolitan secondary schools responded to a questionnaire in three successive years from 2008 to 2010. Participants reported perceptions about their body size and physical appearance and whether they were, or ought to be, on a diet. The data were analysed using a series of longitudinal logistic regression models.

**Results:**

Dieting and dissatisfaction with body size significantly increased over time and more so for older than younger girls. Region significantly moderated the effect of grade level regarding dissatisfaction with body size but not dieting. In non-metropolitan regions, those in the younger cohort were significantly more likely to be dissatisfied with their body size than the older cohort; whereas in metropolitan regions, those in the older cohort were significantly more likely to be dissatisfied with their body size than the younger cohort. Adolescent female’s perceptions of their appearance were unchanged over time, region and grade level.

**Conclusions:**

Differences across time, region and grade level were found among adolescent females on body size and dieting behaviour, but not physical appearance. Adolescent females experience early and increasing body size dissatisfaction and dieting as they age, but stable perceptions of physical appearance. Age and geographic region are important considerations for the timing and targeting of interventions to address body image concerns. Further investigation of regional differences in body image perceptions and factors that affect these is warranted. The findings of this study highlight the ongoing need for strategies during adolescence to promote a healthy appreciation of body size and appearance.

## Background

The nurturing of a healthy body image is a challenge during adolescence [1], particularly for girls [2, 3]. For instance, an Australian survey of 14,461 young people aged 15–19 years found that 42.1 % of adolescent females were concerned about body image and body image was the third major issue of personal concern (behind coping with stress and school/study problems) [4]. Among adolescent females, body dissatisfaction is associated with impaired emotional well-being, low self-esteem, elevated depressive symptoms, low physical activity and disordered eating [5–11]. Importantly, perceptions of body weight and size, rather than actual body mass index (BMI) are more strongly related to these outcomes [8, 9, 12]. Given the detrimental outcomes associated with poor body image, it is important to identify: (a) whether negative body image perceptions tend to decrease over time, as adolescents transition into young adulthood, or whether negative body image remains high; and (b) subgroups that might be particularly vulnerable to experiencing body image concerns. This information will provide direction for research and preventive policy [13].

Two key developmental transitions occur in early to middle adolescence and middle to late adolescence. The examination of body image during these transition periods is imperative as distinct and significant changes occur at these times. During early adolescence, for example, most girls experience marked physical changes associated with puberty and high importance is placed on peer acceptance. In contrast, later adolescence and young adulthood are characterised by fewer physical changes, greater independence, and the challenges of moving into adult environments in tertiary education and the workplace [14, 15].

Research on the body image of adolescent females has predominantly focused on the early to middle adolescent period and has produced conflicting findings. For example, a study of Australian girls in Grades 8 and 10, where data were collected at two time points over 12 months, found a significant decrease in body satisfaction [16]. An earlier 5-year longitudinal study [17] found that body satisfaction decreased for girls between the ages of 13 and 15 years and stabilised between the ages of 15 and 18 years. Another study reported that body satisfaction was stable in a group of 10–15 year old girls followed over a 3-year period [18]. To date, there has been little focus on body image during the older adolescent period and transition to young adulthood. Given the differences in psychosocial development, role expectations, and social pressures between early to middle adolescence and middle to late adolescence, there may be differing body image perceptions [15].

Few studies of body image have tracked young women past completion of secondary school, consequently, there is uncertainty of the unique trajectories of body image perceptions for the period of late adolescence to young adulthood [19]. In one study that has examined transitions into young adulthood, a 10-year study in the US found that body dissatisfaction increased between middle school and high school and increased further during the transition to young adulthood [19]. In contrast, a 5-year longitudinal study [13] reported that adolescent females transitioning from middle school to high school reported a decrease in body satisfaction over time in contrast to slight body satisfaction increases among those transitioning from high school to young adulthood.

There has also been little examination of body image perceptions among the various subgroups within the adolescent female population. In particular, examination of regional differences in body image perceptions among adolescent females is scarce [20, 21]. Compared with adolescents living in urban areas, those living in rural areas have higher rates of overweight and obesity [22, 23]. There is little evidence, however, of whether there are differences in body image perceptions among adolescent females living in different geographical regions. In a prior cross-sectional examination of baseline data from the current study dataset, adolescent females from non-metropolitan regions reported a poorer body satisfaction than those from metropolitan regions [20]. A cross-sectional Canadian study of adolescent females aged 10 and 11 found a similar pattern. That is, after controlling for BMI, girls who resided in rural areas were more likely than urban girls to report poor body satisfaction [21]. Multi-factorial longitudinal research is required to determine if regional differences are present in different age cohorts and elucidate the patterns of change in body image perceptions among different groups of adolescent females.

This study represents an analysis of the Factors Influencing Transitions in Girls’ Active Leisure and Sport (FITGALS) dataset. The present study longitudinally examined the satisfaction with body size and physical appearance for two adolescent female cohorts at transitional phases [14] in two geographic regions (metropolitan and non-metropolitan) over a 3-year period. In this analysis, the first of its kind, we compared the body image of adolescent females in metropolitan and non-metropolitan areas over time. The purpose of this study was to examine (1) Changes over time in body image perceptions of adolescent females, (2) The perceptions of body image for adolescent females in Grade 11 compared to those in Grade 7; and (3) Differences in body image perceptions among girls from non-metropolitan and metropolitan areas.

## Methods

### Study design

This was a 3-wave longitudinal study, with data collected each year from 2008 to 2010. The study was conducted in Victoria, Australia.

### Selection of participants, recruitment and approval

Data for this analysis were from the FITGALS study. The study procedures have been presented elsewhere [20, 24–26] and are briefly described here.

Baseline data were collected for two cohorts of adolescent females, Grade 7 (reflecting transition from young to middle adolescence) and Grade 11 (reflecting transition from secondary school to University or workforce) from Australian metropolitan and non-metropolitan secondary schools using stratified random sampling. Ethical approval for this study was received from the relevant Universities, the Victorian Department of Education and the Victorian Catholic Education Office.

### Measure of body image

This study adopted the definition of body image posited by Meland and colleagues [27], namely body image is “the individual, subjective sense of satisfaction or dissatisfaction with one’s body or physical appearance” (p. 343). Accordingly, measures were based on those used by Meland et al. [27]. Participants were asked three questions about satisfaction/dissatisfaction of their physical size and appearance and dieting behaviour (with the response options in parentheses), namely (a) ‘Do you think your body is …’ (‘much too thin’, ‘a bit too thin’, ‘about the right size’, ‘a bit too fat’, ‘much too fat’, ‘I don’t think about it’); (b) ‘Do you think you are…’ (‘very good looking’, ‘quite good looking’, ‘about average’, ‘not very good looking’, ‘not at all good looking’, ‘I don’t think about my looks’) and (c) ‘Are you on a diet to lose weight?… (‘no, because my weight is fine’, ‘no, but I need to lose weight’, ‘yes’). These variables are similar to those which have demonstrated adequate validity in the literature on body image disturbances [28]. Dieting is often examined as a separate construct in this literature. However, the concept of body image pertains to an individual’s perception, attitudes about the perception, as well as associated behaviours [27].

### Statistical analysis

Data screening was undertaken prior to conducting the data analyses. All analyses were conducted using SPSS Version 21. While the categorical data were broadly ordinal in nature, it was considered that in each question the relationships between categories were more complex than a simple one-dimensional continuum. Consequently the data were recast as a number of dichotomies, each of which was separately analysed using longitudinal logistic regression models fitted by the method of Generalised Estimating Equations (GEE). For perceptions of body size, the ‘I don’t think about it’ category was excluded from the analyses due to ambiguity of meaning and an inability of the researchers to follow this up with participants. The ‘too fat and ‘much too fat’ categories were collapsed into ‘too fat’ and the ‘too thin’ and ‘much too thin’ categories were collapsed into ‘too thin’. Two separate analyses were conducted (comparing ‘too fat’ to ‘about right’, and ‘too thin’ to ‘about right’), because issues around ‘too thin’ (i.e., possible anorexia/bulimia) are possibly different to those around ‘too fat’ (i.e., obesity), meaning that ‘too fat’ and ‘too thin’ could not be collapsed into a general ‘dissatisfied’ (‘too fat’ or ‘too thin’) category. Thus body size was analysed using three categories, but in two separate analyses.

Examination of physical appearance was collapsed into ‘good looking’, ‘about average’, or ‘not good looking’ (and the ‘I don’t think about it’ category was excluded from the analyses for reasons outlined in the previous paragraph). Two separate analyses were conducted (comprising ‘better looking than average’ compared to ‘average looking’ and ‘less than average looking’ compared to ‘average looking’). The response categories for the question about whether participants were currently on a diet (‘no, because my weight is fine’, ‘no, but I need to lose weight’, ‘yes’) remained as three options. Two separate analyses were conducted (‘no, because my weight is fine’ compared to ‘yes’, and ‘no, but I need to lose weight’ compared to ‘yes’).

For each of the dichotomies described GEE models were used to identify whether there were significant differences between the proportions of responses between grade levels at recruitment [13, 19], regions (metropolitan, non-metropolitan) and years (2008, 2009, 2010). In each case, the possibility of correlation in the longitudinal data was tested using four correlation structures of increasing complexity (independence, exchangeable, first order auto-regressive and unstructured). In all cases, the simplest independence model was judged to be appropriate. Each model was tested for main effects (grade level, region and year) and interaction effects, but if no interaction effects were significant, the model was reanalysed for main effects alone.

## Results

### Sample characteristics

Of the 732 participants recruited in 2008, 71.2 % (*n* = 521) were from metropolitan schools and 28.8 % (*n* = 211) were from non-metropolitan schools; 66.8 % (*n* = 489) were first recruited in Year 7 at secondary school and the remainder (*n* = 243, 33.2 %) were initially recruited in Year 11. The retention rates in 2009 and 2010 were 82.4 % (Year 7 83.6 %, Year 11 80.7 %) and 74.0 % (Year 7 81.9 %, Year 11 57.7 %) respectively. Similar retention rates were observed between metropolitan (62 %) and non-metropolitan (59 %) regions. Table 1 shows the number of participants in relation to grade level and region over the 3 year study.

**Table 1:** Number of participants in each year of the study (and the percentage of participants retained, compared to the first year of the study): by region and grade level at recruitment

Year	Region	Grade level at recruitment	
		Grade 7	Grade 11
2008	Metropolitan	366	155
	Non-metropolitan	123	88
2009	Metropolitan	310 (85 %)	123 (79 %)
	Non-metropolitan	99 (80 %)	73 (83 %)
2010	Metropolitan	253 (69 %)	70 (45 %)
	Non-metropolitan	81 (66 %)	44 (50 %)

At baseline, the majority of participants were aged 11–13 (*n* = 701, M ± SD = 13.6 ± 1.98). Participants who returned all three survey forms comprised: Grade 7 (*n* = 328, 74.5 %; aged 11–13, M ± SD = 12.2 ± 0.5 years at baseline) and Grade 11 (*n* = 112, 25.5 %; aged 16–18, 16.2 ± 0.6 years at baseline).

### Perception of body size

Table 2 summarises the patterns of responses regarding perceived body size, within each combination of year, grade level and region. Table 2 shows that overall, across the 3 years of the study, only 8 % identified themselves as being ‘too thin’, 56 % reported that they were ‘about right’ and 37 % reported that they were ‘too fat’.

**Table 2:** Frequencies (and percentages) of participants’ reports of body size: by region within grade level at recruitment within year

Year	Grade level at recruitment	Region	‘Too thin’		‘About right’		‘Too fat’		Total	
			*n*	%	*n*	%	*n*	%	*n*	%
2008	7	Metropolitan	33	10.1	198	60.6	96	29.4	327	100.0
		Non-metropolitan	7	6.0	65	55.6	45	38.5	117	100.0
	11	Metropolitan	9	6.3	83	57.6	52	36.1	144	100.0
		Non-metropolitan	3	3.6	52	62.7	28	33.7	83	100.0
2009	7	Metropolitan	22	7.6	171	59.0	97	33.4	290	100.0
		Non-metropolitan	8	8.7	42	45.7	42	45.7	92	100.0
	11	Metropolitan	12	10.4	57	49.6	46	40.0	115	100.0
		Non-metropolitan	2	3.0	44	65.7	21	31.3	67	100.0
2010	7	Metropolitan	20	8.3	129	53.8	91	37.9	240	100.0
		Non-metropolitan	6	7.7	33	42.3	39	50.0	78	100.0
	11	Metropolitan	8	12.1	26	39.4	32	48.5	66	100.0
		Non-metropolitan	2	4.5	24	54.5	18	40.9	44	100.0
Total			132	7.9	924	55.6	607	36.5	1663	100.0

**Fig. 1:**
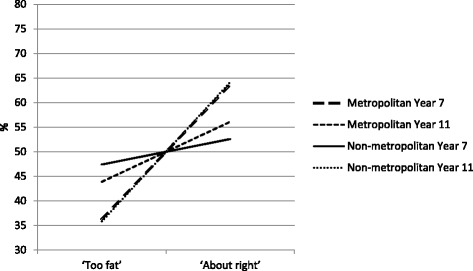
Percentage of participants’ reports of body size; aggregated over years

For comparisons of ‘too fat’ versus ‘about right’, there was a statistically significant main effect for year (Wald Chi-square (2) = 14.548, *p* = 0.001), but not for region, nor grade level. A girl was 46 % more likely to classify herself as being ‘too fat’ in 2010 than in 2008 (Odds Ratio (OR) = 1.455, 95 % confidence interval (CI), 1.094–2.071). There was a statistically significant interaction between region and grade level (Wald Chi-Square (1) = 6.705, *p* = 0.010) irrespective of year of study, with the difference in the likelihood of Grade 7 compared to Grade 11 girls identifying themselves as being ‘too fat’ being 64 % less for metropolitan girls than for non-metropolitan girls (OR = 0.364, 95 % CI = 0.139–0.953), consistently across the 3 years of the study. Further odds ratio calculations (see Table 3) revealed that in the metropolitan sample, Grade 7 girls were less likely than Grade 11 girls to perceive themselves as being too fat (OR = 0.728, 95 % CI = 0.555–0.956), whereas in the non-metropolitan sample, the reverse was the case (OR = 1.612, 95 % CI = 1.098–2.366; See Fig. 1). For comparisons of ‘too thin’ with ‘about right’, there were no statistically significant differences for year, region or grade level, and no significant interactions.

**Table 3:** Frequencies of participants’ reports of body size; aggregated over years and showing odds ratios (OR) for grade levels within regions

Region	Grade level at recruitment	‘Too fat’		‘About right’		Odds	OR	95 % CIs
		*n*	%	*n*	%			
Metropolitan	7	284	36.3	498	63.7	0.57	0.73	0.555–0.956
	11	130	43.9	166	56.1	0.78		
Non-metropolitan	7	126	47.4	140	52.6	0.90	1.61	1.098–2.366
	11	67	35.8	120	64.2	0.56

In summary, no differences across time, region or grade level were found among girls identifying with being ‘too thin’. However, over time girls increasingly perceived themselves as ‘too fat’, Grade 7 metropolitan girls were less likely to think of themselves as being ‘too fat’ than Grade 11 metropolitan girls, and Grade 7 non-metropolitan girls were more likely to think of themselves being ‘too fat’ than Grade 11 non-metropolitan girls.

### Perception of physical appearance

Table 4 summarises the patterns of responses regarding physical appearance, within each combination of year, grade level and region. Table 4 shows that overall, across the 3 years of the study, 26 % reported that they were above average in appearance, 58 % thought they were of average appearance, and 17 % thought they were below average in appearance.

**Table 4:** Frequency (and percentage) of participants’ reports of physical appearance: by region within grade level at recruitment within year

Year	Grade level at recruitment	Region	‘Better looking than average’		‘About average’		‘Worse looking than average’		Total	
			*n*	%	*n*	%	*n*	%	*n*	%
2008	7	Metropolitan	106	35.5	155	51.8	38	12.7	299	100.0
		Non-metropolitan	24	26.1	51	55.4	17	18.5	92	100.0
	11	Metropolitan	32	25.2	71	55.9	24	18.9	127	100.0
		Non-metropolitan	24	33.8	41	57.7	6	8.5	71	100.0
2009	7	Metropolitan	67	25.9	143	55.2	49	18.9	259	100.0
		Non-metropolitan	16	19.5	52	63.4	14	17.1	82	100.0
	11	Metropolitan	21	24.1	52	59.8	14	16.1	87	100.0
		Non-metropolitan	17	27.4	40	64.5	5	8.1	62	100.0
2010	7	Metropolitan	63	28.8	120	54.8	36	16.4	219	100.0
		Non-metropolitan	10	13.9	42	58.3	20	27.8	72	100.0
	11	Metropolitan	12	7.1	120	71.4	36	21.4	168	100.0
		Non-metropolitan	17	41.5	22	53.7	2	4.9	41	100.0
Total			409	25.9	909	57.6	261	16.5	1579	100.0

Note: There were no statistically significant main effects for year, region or grade level, and no significant interactions

Analysis of participants’ responses about appearance revealed no statistically significant main effects for year, region or grade level, and no significant interactions, regarding those who identified themselves as ‘better looking than average’ versus those identified themselves as being ‘about average looking’ nor regarding those identified as ‘worse looking than average’ compared with ‘average looking’.

In summary, girls’ reports of how good looking they were (in comparing ‘better looking than average’ to ‘about average looking’, or between ‘worse looking than average’ with ‘average looking’) did not differ from year to year, between grade levels or between regions.

### Dieting behaviour

Table 5 summarises the patterns of responses regarding dieting behaviour, within each combination of year, grade level and region. Table 5 shows that overall, across the 3 years of the study, 57 % of participants were not on a diet and thought their weight was fine, 11 % were on a diet, and 32 % were not on a diet but felt that they needed to lose weight.

**Table 5:** Frequencies (and percentages) of responses to the question ‘Are you on a diet?: by region within grade level within year

Year	Grade level at recruitment	Region	‘Not on a diet because I don’t need to lose weight’		‘Yes, I’m on a diet’		‘Not on a diet but needing to lose weight’		Total	
			*n*	%	*n*	%	*n*	%	*n*	%
2008	7	Metropolitan	245	69.0	18	5.1	92	25.9	355	100.0
		Non-metropolitan	79	65.3	5	4.1	37	30.6	121	100.0
	11	Metropolitan	84	38.4	52	23.7	83	37.9	219	100.0
		Non-metropolitan	49	53.8	17	18.7	25	27.5	91	100.0
2009	7	Metropolitan	184	59.7	30	9.7	94	30.5	308	100.0
		Non-metropolitan	54	55.1	5	5.1	39	39.8	98	100.0
	11	Metropolitan	62	51.2	17	14.0	42	34.7	121	100.0
		Non-metropolitan	43	62.3	9	13.0	17	24.6	69	100.0
2010	7	Metropolitan	137	55.2	34	13.7	77	31.0	248	100.0
		Non-metropolitan	43	53.1	5	6.2	33	40.7	81	100.0
	11	Metropolitan	35	50.0	11	15.7	24	34.3	70	100.0
		Non-metropolitan	25	55.6	4	8.9	16	35.6	45	100.0
Total			1040	57.0	207	11.3	579	31.7	1826	100.0

Note: Statistically significant main effects for year (Wald Chi-Square (2) = 15.020, *p* = 0.001) and grade level (Wald Chi-square (1) = 10.001, *p* = 0.002) but not for region in comparison between participants on a diet and those who were not on a diet because their weight was fine. There were no significant interactions

Statistically significant main effect was found for grade level (Wald Chi-Square (1) = 5.948, *p* = 0.015), but not for year, nor for region, in comparison between those on a diet compared to those who were not on a diet but did need to lose weight. There were no significant interactions

In the comparison between participants who reported they were ‘on a diet’ and those who said they were not on a diet because their weight was fine, statistically significant main effects were found for year (Wald Chi-Square (2) = 15.020, *p* = 0.001) and grade level (Wald Chi-square (1) = 10.001, *p* = 0.002) but not for region. The odds of a girl saying she was on a diet were 56 % higher in 2009 than in 2008 (OR = 1.563, 95 % CI = 1.121–2.180), and 109 % higher in 2010 than 2008, (OR = 2.089, 95 % CI = 1.429–3.056) irrespective of region and grade level. The odds of a girl recruited in Grade 11 being on a diet were 97 % higher than a girl recruited in Grade 7 (OR = 1.973, 95 % CI = 1.295–3.007), irrespective of region and year. There were no significant interactions.

In analysing the reports of those who were ‘on a diet’ compared to those who said they were not on a diet but did need to lose weight a statistically significant main effect was found for grade level (Wald Chi-Square (1) = 5.948, *p* = 0.015), but not for year, nor for region. The odds of a girl recruited in Grade 11 classifying herself as ‘on a diet’ rather than ‘not on a diet but needing to lose weight’ were 64 % higher than a girl recruited in Grade 7 (OR = 1.642, 95 % CI = 1.102–2.446). There were no significant interactions.

In summary, the likelihood of girls being on a diet rather than not on a diet or not on a diet but needing to lose weight increased over time, and older girls were more likely to be on a diet than younger girls. Region was not a factor relating to dieting behaviour.

## Discussion

Poor body image is a significant public health issue, particularly among adolescent females [4] and is associated with a range of detrimental outcomes [5–7, 11]. Our findings contribute to our understanding of body image by examining patterns in body image perceptions of adolescent females representing two different transition periods and two different geographic regions. We found that (a) dissatisfaction with body size increased over time for girls recruited in Grade 7 and Grade 11 in both metropolitan and non-metropolitan regions; (b) in non-metropolitan regions, girls in the younger cohort were more likely to be dissatisfied with their body size than the older cohort; whereas in metropolitan regions, girls in the older cohort were more likely to be dissatisfied with their body size than the younger cohort; (c) satisfaction with physical appearance did not change over time for Grade 7 and Grade 11 girls in either metropolitan or non-metropolitan regions, with no significant differences between the older and younger girls in perceptions of their appearance; and (d) Grade 7 and Grade 11 girls in both metropolitan and non-metropolitan areas reported more dieting over time with older girls more likely to report being on a diet than younger girls.

Our findings suggest that perceptions of body size are dynamic, with more girls considering that they are ‘too fat’ progressively over time. This finding supports previous findings of increasing body dissatisfaction during adolescence [16, 19]. In our study, there were consistently higher proportions of Grade 11 females reporting being ‘too fat’ than Grade 7s, with 45.5 % of the older group compared to 40.9 % of the younger girls reporting being too fat at the end of the survey period. To date, most of the research on body image has focused on younger adolescent females. The current study adds to the literature by suggesting that older adolescent females transitioning to young adulthood also experience increasing body dissatisfaction, which is consistent with the few studies of older adolescents, such as that of Bucchianeri et al. [19]. Older girls may have more discussions with peers, and make more social comparisons, about body size than their younger counterparts [29]. It is also possible that older girls are more readily influenced by Western ideals of beauty that often depict unattainable benchmarks against which girls evaluate their bodies and those of others [30].

A novel aspect of our study was the comparison between metropolitan and non-metropolitan adolescents across two age cohorts. We found an interaction effect of grade level and region whereby in the metropolitan sample, younger adolescent females were *less* likely than older adolescent females to perceive themselves as being too fat, whereas in the non-metropolitan sample, the younger adolescent females were *more* likely than the older adolescent females to perceive themselves as being too fat. A cross sectional Canadian study of girls aged 10 and 11 (similar in age to our younger cohort), found that girls who resided in rural areas were more likely than urban girls to report poor body satisfaction (controlling for BMI). Our study adds to the existing literature on regional differences in body image perceptions by suggesting that regional differences in body satisfaction might vary according to stage in adolescence. This requires further investigation as it has implications for the targeting and timing of interventions to promote positive body image during adolescence. Additional research is needed to clarify how body size related norms and pressures differ by geographic region and the influence of family and peers on body image perceptions.

Regarding satisfaction with physical appearance, we did not find any differences over time, between age groups or between regions. It is somewhat surprising that we did not find differences between the younger and older cohorts or changes over time, since previous research has suggested that older adolescents tend to perceive themselves as less good looking than do younger adolescents. For example, Meland et al. [27] found girls aged 13 and 15 years considered they are ‘not as good looking’ as those aged 11 years and Young and Mroczek [31] reported that older adolescents had lower physical appearance satisfaction than younger adolescents. Notwithstanding, our findings support the notion that perceptions about physical appearance appear in early puberty [32], and, for Australian girls, these perceptions do not change through adolescence. Our findings may further indicate girls’ understanding and acceptance that physical looks cannot readily be changed in contrast to body size that can be more easily changed with dieting, more exercise and/or a change in eating and life-style regimes.

In relation to dieting, our study found that girls in both metropolitan and non-metropolitan areas dieted, and increasingly did so as they got older. For example, in the first year of data collection 22.3 % of Grade 11 girls were on a diet and a further 34.8 % were not on a diet but thought that they needed to lose weight. This finding is consistent with another of our key findings that girls were increasingly dissatisfied with their body size as they aged. Indeed, the literature supports a strong link between dieting and body dissatisfaction with efforts to control weight intensifying as girls become increasingly displeased with their physique [11, 33]. The findings of the present study on dieting behaviour are consistent with the comparison between older and younger girls by Meland et al. [27]. This consistency suggests there may be cultural similarities between Australia and Norway in girls’ responses as they move away from the thin-ideal and, in many instances, receive less social support from parents than in earlier years.

Recent research on the factors that influence negative body image perceptions has focused on the role of the social media. Evidence suggests that the use of social media has a negative influence on body image [34–36], which is concerning given the high use of the internet and social media sites by adolescents. A recent survey of adolescents in Australia showed that the internet was ranked as their main source of information by 72.8 % of respondents, and 21.3 % reported that they spend at least 20 h a week on social networking sites [4]. The prevalence of social media and its impact on body image perceptions underscores the importance of interventions to promote positive body image during adolescence.

Body image is an important public health issue and body image perceptions can be modified through intervention [37, 38]. For example, *Happy Being Me* is a prevention program designed specifically for early secondary school girls which has demonstrated positive outcomes [38]. This approach is consistent with our study’s findings that targeting girls in early adolescence is appropriate. Intervention in early adolescence might be particularly important in non-metropolitan areas, with these girls experiencing high levels of body dissatisfaction. The results of our study further suggest that ongoing intervention is be required during older adolescence and transition to young adulthood. Thus, in addition to school programs, there are potential roles for government, parents, coaches, and the media to help girls to develop healthy appreciation of their unique attributes and qualities. It is imperative that girls are resilient [39] and discerning in the face of multiple cultural influences that define beauty in terms of ‘thinness’ [40, 41].

Our study has a number of strengths. Firstly, the study’s design facilitated an examination of body size, physical appearance and dieting at critical developmental transitions for girls living in different geographical regions. Secondly, the study it as one of a limited number of longitudinal studies of body image in both younger and older adolescent females, and as such, enhances the ability to extrapolate trends, patterns and trajectories over time. It is, however, noted that truly valid comparisons of trends identified in longitudinal studies are challenging given the range of measures to assess body image that have been adopted to date. In this context, a strength of our study is its examination of key body image variables - perceptions of body size and physical appearance and dieting behaviour – that are theoretically derived [27].

Our study has several limitations. Firstly, the study is primarily descriptive and did not examine the processes behind changes in body image. It is recommended that future studies examine processes of changes and consider collecting data from a variety of sources (e.g., parents, teachers). The low retention rate in this study’s third year suggests a self-selection bias and, given that schools were the primary sampling units, the samples are unlikely to be representative of all adolescent females. Avoiding self-selection bias and retaining participants in longitudinal cohort studies is a challenge. A range of strategies were used in this study to address the potential for self-selection bias and to aid in the retention of study participants. Briefing notes were provided to teachers, which stressed the importance placed by the researchers on the experiences and opinions of all girls and small incentive prizes were provided to study participants. School staff played a crucial role in the promotion, recruitment and retention of participants. In recognition of this important role, the coordinating teacher in each school received a small gift voucher and participating schools received a voucher for the purpose of equipment purchase. There was a particularly low retention rate in Year 13 (Year 11, Wave 3), this was likely due to the lack of school support and motivation for participants post-schooling, as well as the difficulty of obtaining accurate contact details in advance of Wave 3.

## Conclusion

In summary, this study found differences across time, region and grade level among adolescent females regarding perceptions of body size and dieting behaviour, but not perceptions of physical appearance. Adolescent females experience early and increasing body size dissatisfaction and dieting as they age, but stable perceptions of physical appearance. The findings of the present study support early intervention to promote positive body perceptions, starting in childhood and continuing in to adulthood and examination of geographic region as a potential factor when examining patterns and trends in body image perceptions among adolescent females.

## Abbreviations

BMI: Body mass index
CI: Confidence interval
FITGALS: Factors influencing transitions in girls’ active leisure and sport
GEE: Generalised estimating equations
M: Mean
OR: Odds ratio
